# *Kymachrysa*, a new genus of Nearctic Green Lacewings (Neuroptera, Chrysopidae, Chrysopini)

**DOI:** 10.3897/zookeys.437.7984

**Published:** 2014-08-28

**Authors:** Catherine A. Tauber, J. Allan Garland

**Affiliations:** 1 Department of Entomology, Comstock Hall, Cornell University, Ithaca, NY 14853 and Department of Entomology & Nematology, University of California, Davis, CA 95616, U.S.A.; 2 Department of Entomology, Macdonald College of McGill University, Ste. Anne de Bellevue, Quebec, Canada H9X 3V9 and P. O. Box 24143, Penticton, British Columbia V2A 8L9, Canada

**Keywords:** New genus, adult, larva, biology

## Abstract

Two North American species of green lacewings have undergone a number of changes in their generic assignments and are currently classified as *incertae sedis*. Here we demonstrate that adults (both sexes) and larvae of these species share a set of features that distinguishes them from currently described genera. Thus, to promote nomenclatural stability in Chrysopidae, we describe *Kymachrysa*, a **gen. n.** that contains the two species – *Kymachrysa intacta* (Navás), **comb. n.** and *Kymachrysa placita* (Banks), **comb. n..** Also, we present modifications for the current generic-level key, illustrations, as well as biological information for identifying the genus and its known species.

## Introduction

The green lacewing tribe Chrysopini (Neuroptera: Chrysopidae) includes seventeen genera in the New World; ten of these occur in the Nearctic. These New World genera are distinguished from each other on the basis of both adult (male and female), as well as larval, characters (e.g., see Brooks and Barnard 1990; Tauber 1974, 1975, 2003; Adams and Garland 1982; Brooks 1994; Penny et al. 2000; Tauber and de Leon 2001; Tauber et al. 2000, 2014; Freitas and Penny 2001). However, the adult and larval characteristics of two Nearctic species were shown to be inconsistent with the genera to which the species had been assigned (*Ceraeochrysa* and *Chrysopodes*), nor could they be assigned to any other known chrysopine genus (Tauber and Flint 2010: 64). Most recently, they were retained temporarily in *Ceraeochrysa* (with the caveat of *incertae sedis*) (Tauber and Flint 2010: 64). Here, to foster nomenclatural stability, we describe a new genus to accommodate these two species (original names: *Chrysopa placita* Banks and *Chrysopa intacta* Navás), and we provide information for their identification.

## Specimens and methods

Our procedures were those used in previous publications (e.g., Tauber et al 2012, Silva et al. 2013). We examined specimens from the following collections: BMNH, The Natural History Museum (formerly British Museum of Natural History), London, England; CAS, California Academy of Sciences, San Francisco, CA; CMNH, Carnegie Museum of Natural History, Pittsburgh, PA; CNC, Canadian National Collection, Ottawa, Canada; CPG, C. P. Gilette Museum of Arthropod Diversity, Colorado State University, Fort Collins, CO; CUIC, Cornell University Insect Collection, Ithaca, NY; EMEC, Essig Museum of Entomology, University of California, Berkeley, CA; MCZ, Museum of Comparative Zoology, Harvard University, Cambridge, MA; ROM, Royal Ontario Museum, Toronto, ON, Canada; SDNHM, San Diego Natural History Museum, San Diego, CA; TRC, M. J. & C. A. Tauber Research Collection, Davis, CA; USNM, National Museum of Natural History (formerly United States National Museum), Smithsonian Institution, Washington, D.C.

## Systematics

### Genus Kymachrysa gen. n.

Taxon classificationAnimaliaNeuropteraChrysopidae

http://zoobank.org/56199711-0065-45BA-A035-414069FFA32D

#### Type-species.

*Chrysopa placita* Banks, 1908: 259.

#### Distinguishing adult features.

*Kymachrysa* adults appear to be typical chrysopine lacewings of medium size and green coloration. Their most distinctive adult features occur in the male and female terminalia; in addition, a few external features are diagnostic of the genus:

#### External features.

**Figure 1.**
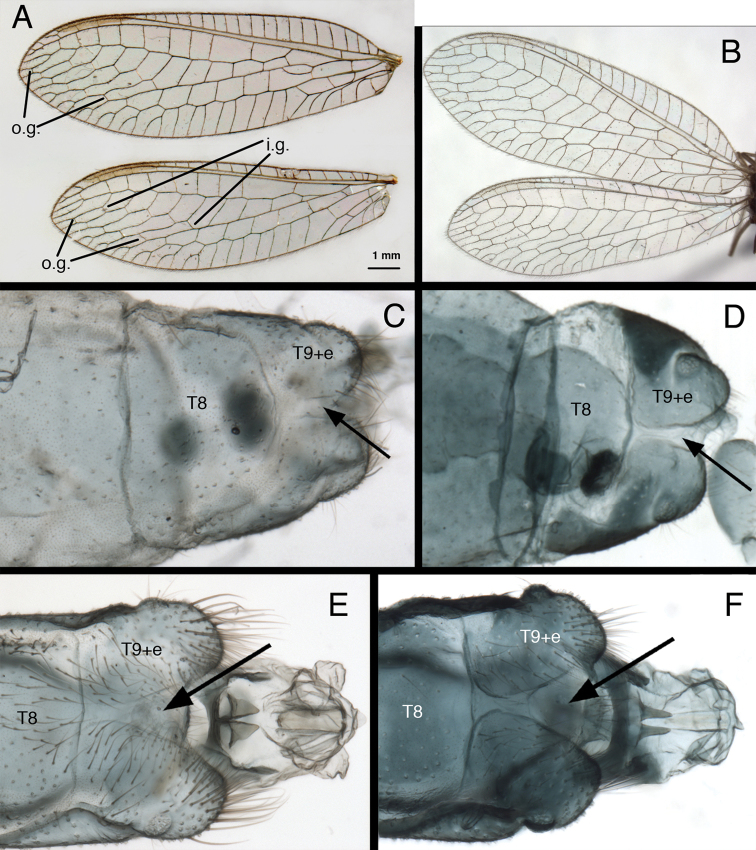
Two external features that characterize *Kymachrysa* adults. **A, B** Fore and hind wings with sinuate longitudinal veins between the first and second gradate series of crossveins **C–F** Terminal segments (dorsal) with Tergite 9+ectoproct separated dorsally **C, D** Female **E, F** Male **A, C, E.** *Kymachrysa placita* **A, C** Colorado, USNM **E** Type, Colorado, MCZ **B, D, F** *Kymachrysa intacta* **B** Neotype, Quebec, CNC **D** New York, TRC **F** New York, TRC [Males: gonarcal complex not removed]. *Abbreviations*: **i.g.** inner gradate veins **o.g.** outer gradate veins **T8** eighth tergite **T9+e** fused ninth tergite and ectoproct.

(i) The longitudinal (radial) veins between the first and second rows of gradate veins of the fore and hind wings are sinuous (Fig. 1A, B), whereas in most other chrysopid genera they are relatively straight. (ii) In both males and females, the fused ninth tergite and ectoproct is completely divided by a dorsal invagination, and each side of the terminal abdominal segment is rounded posterolaterally (especially in males) (Fig. 1C–F). Among other New World genera, a complete dorsal invagination of the T9+ect is reported only for *Chrysopiella* and *Parachrysopiella* (Brooks and Barnard 1990).

#### Male terminalia.

**Figure 2.**
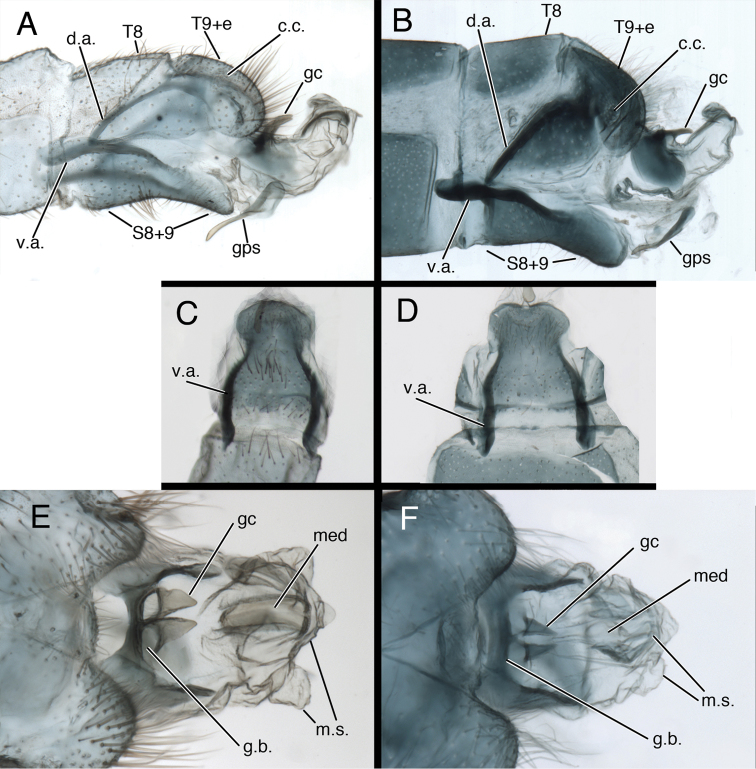
*Kymachrysa* male terminalia. **A, B** Abdominal segments 8 and 9 (lateral), with genitalia everted **C, D** Sternite 8+9 (ventral) **E, F** Genitalia (everted, dorsal) **A, C, E** *Kymachrysa placita* Type, Colorado, MCZ **B, D, F** *Kymachrysa intacta*, California, TRC. *Abbreviations*: **c.c.** callus cerci **d.a.** dorsal apodeme (on ninth tergite and ectoproct) **gc** gonocornu **gps** gonapsis **g.b.** gonarcal bridge **med** mediuncus **m.s.** membranous sac **S8+9** fused eighth and ninth sternites **T8** eighth tergite **T9+e** fused ninth tergite and ectoproct **v.a.** ventral apodeme (on sternite 8+9).

One of the most striking aspects of the *Kymachrysa* male terminalia is the S8+9, which is entirely fused and well sclerotized as in most chrysopine genera. However, in lateral view, the *Kymachrysa* S8+9 has an unusual ventral bend, and the ventral apodeme is heavy and elongate – extending anteriorly well beyond the proximal margin of S8 (Fig. 2A, B). In ventral view, S8+9 is constricted mesally and rounded both anteriorly and posteriorly (Fig. 2C, D). These features are unique among New World chrysopids.

Gonocornua are present on the gonarcal bridge, as in *Ceraeochrysa*. In *Ceraeochrysa*, the gonocornua are usually rounded and unarticulated, and they arise laterally from the gonarcal bridge (see Freitas et al. 2009). But, the gonocornua of *Kymachrysa* are unusual in that they appear at least partially articulated or separated from the gonarcal bridge, and at the base they are juxtaposed and located mesally on the distal margin of the gonarcal bridge (Fig. 2E, F). Furthermore, the dorsum of the mediuncus has a distinct, trough-like shape not found in other genera; the terminus has a weak, lightly sclerotized or membranous beak mesally and expanded membranous sacs laterally.

#### Female terminalia.

**Figure 3.**
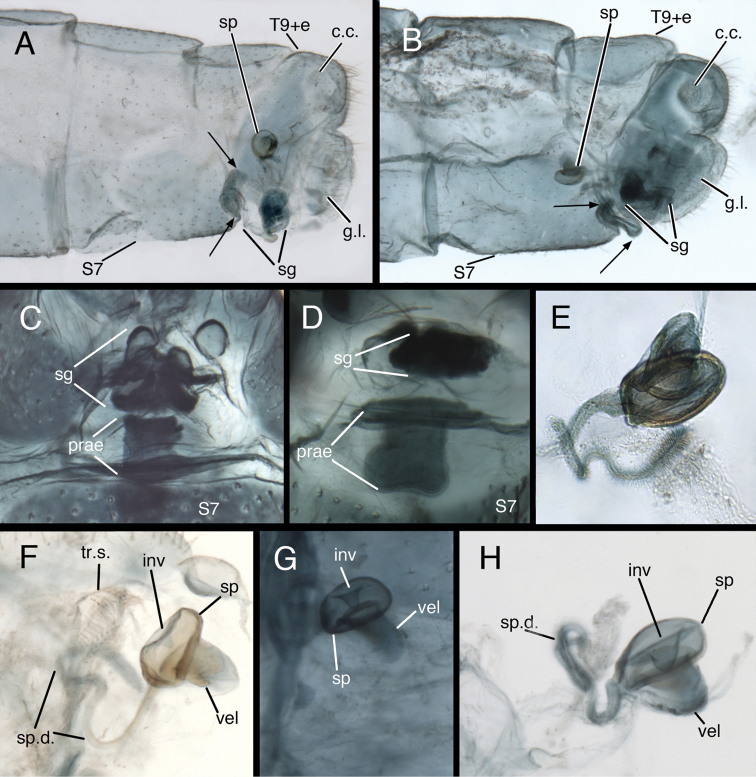
*Kymachrysa* female terminalia. **A, B** Abdominal segments 7 and 9 (lateral) [Arrows delineate the praegenitale] **C, D** Praegenitale at distal margin of S7 (ventral) **E–H** Spermatheca **A, C, F** *Kymachrysa placita* **A, F** Colorado, USNM **C** New Mexico, USNM **B, D, E, G, H** *Kymachrysa intacta* **B, D, E** New York, TRC **G** Colorado, CPG **H** California, TRC. *Abbreviations*: **c.c.** callus cerci **g.l.** gonapophysis lateralis **inv** spermathecal invagination **prae** praegenitale **sg** subgenitale **sp** spermatheca **sp.d.** spermathecal duct **S7** seventh sternite **tr.s.** transverse sclerite **T9+e** fused ninth tergite and ectoproct **vel** velum.

Two notable features distinguish the female genitalia. (i) This chrysopid genus is the only one outside of the tribe Belonopterygini (see Tjeder 1966: 235, 324, 337) in which the female is reported to have a praegenitale. [Note: the recording of a praegenitale for *Leucochrysa* (data matrix of Brooks and Barnard 1990: Table 1) appears to be an error – the description of the genus, p. 248, states that the structure is absent.] Moreover, the structure appears unique among chrysopids, in that it is asymmetrical (a condition not reported for Belonopterygini) (Fig. 3C, D). (ii) The spermatheca is shaped like a pillbox with a shallow invagination and a sail-shaped velum that opens via a slit to the bursal duct (Fig. 3F–H). By comparison, in *Ceraeochrysa* the spermatheca is cylindrical, with an elongate invagination and a U-shaped or J-shaped bend that opens via a slit directly to the bursa copulatrix (e.g., Adams and Penny 1985, Freitas and Penny 2001, Sosa and Freitas 2010, 2011). And, in *Chrysopodes* the spermatheca is cylindrical or tubular, with a very deep invagination and an elongate bursal duct (e.g., Adams and Penny 1985, Freitas and Penny 2001, Tauber 2010, Tauber et al. 2012). (iii) Finally, the *Kymachrysa* spermathecal duct (mature specimens) is hairy for almost its entire length; the terminal bristles are long and fine, and their length decreases proximally; at the base of the duct the bristles are very short and stubby, or granular in appearance (Fig. 3E, F).

#### Description.

**Figure 4.**
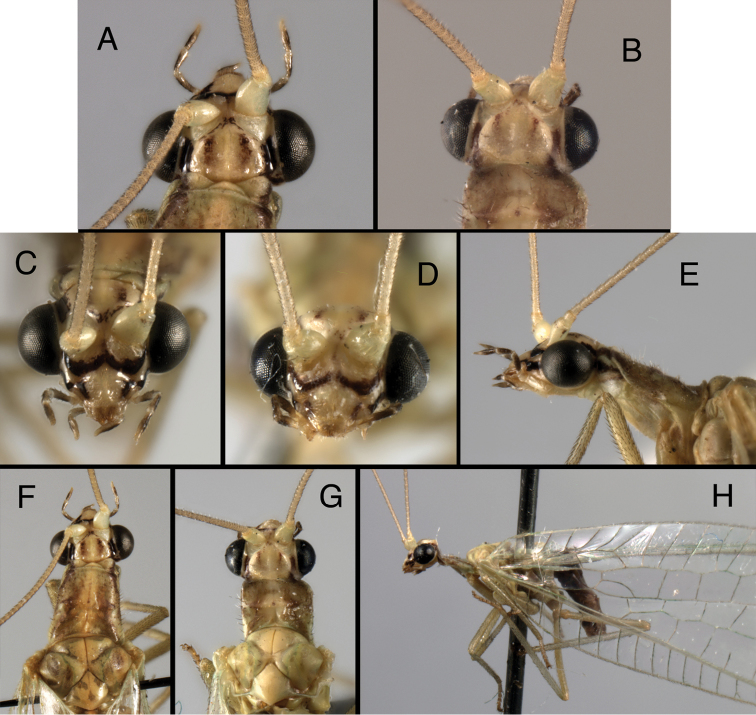
*Kymachrysa placita* external adult features. Note variation in darkness and size of head markings, prothoracic size and shape. **A, B** Head (dorsal) **C, D** Head (frontal) **E** Head, prothorax (lateral) **F, G** Head, prothorax, mesothorax (dorsal) **H** Head, thorax (lateral) [Background with small piece of abdomen visible]. **A, C, E, F** Wyoming, SDNHM; **B, D, G, H** Colorado, CPG.

**Figure 5.**
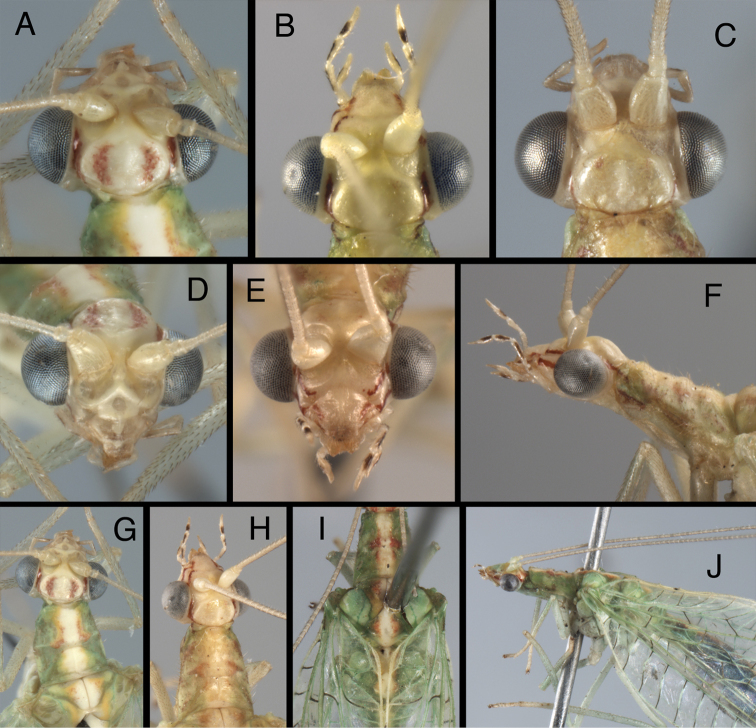
*Kymachrysa intacta* external adult features. Note variation in the presence, darkness, and size of head and thoracic markings. **A–C** Head (dorsal) **D, E** Head (frontal) **F** Head, prothorax (lateral) **G, H** Head, prothorax, mesothorax (dorsal) **I** Thorax (dorsal) **J** Head, thorax (lateral) **A, D, G, I, J** California, TRC **B, E, F, H** Colorado, TRC; **C** New York, TRC.

**Adult** (Figs 4, 5, 8C, D). Delicate, slender, medium sized (forewing length, *Kymachrysa placita*: 11–13 mm; *Kymachrysa intacta* 11–15 mm), predominantly green with yellow longitudinal stripe, mesally. **Head** (Figs 4, 5). Vertex with pair of crescent-shaped, red or brown sublateral marks (sometimes absent or faint), usually with lateral red or brown stripe near margin of eye; frons with or without markings; gena with red or brown longitudinal markings. Distal segments of labial, maxillary palpi with elongate, lateral, black marks. Antenna cream colored, without markings. **Thorax** (Figs 4E–H, 5F–J, 8C, D). Prothorax variable in length and shape (probably developmental variation), usually long, tapered distally; dorsum without lateral stripes, but usually with irregular, red or brownish, sublateral markings (especially western, southern specimens); mesothorax, metathorax with or without markings. Legs mostly light green, without markings, with numerous dark brown to black setae; tarsal claws with deep U-shaped to V-shaped cleft.

Wings (Fig. 1A, B) with costal area slightly enlarged basally; radius straight; im3 cell triangular; forewing, hindwing with two, regular, slightly converging rows of gradate veins; longitudinal veins between inner and outer gradates sinuate; three icu cells, distal one open.

**Figure 6.**
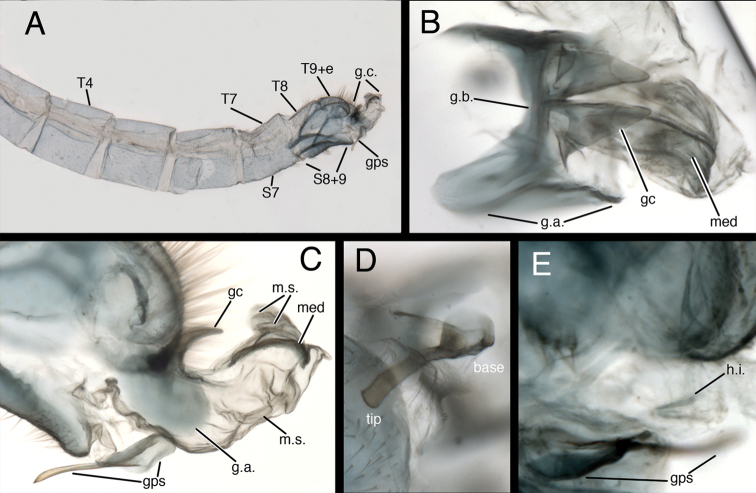
*Kymachrysa placita* male abdomen. **A** Abdominal segments 3 to terminus (lateral), with genitalia everted **B** Gonarcal complex (dorsal) **C** Genitalia (everted–including gonapsis, lateral) **D** Gonapsis (ventral) **E** Hypandrium internum nestled within membrane [Gonapsis not in focus, shown for relative scale]. All Types, Colorado, MCZ *Abbreviations*: **gc** gonocornu **gps** gonapsis **g.a.** gonarcal apodeme **g.b.** gonarcal bridge **g.c.** gonarcal complex **h.i.** V-shaped hypandrium internum **med** mediuncus **m.s.** membranous sac **S7** seventh sternite **S8+9** fused eighth and ninth sternites **Tx** tergite, number **T9+e** fused ninth tergite and ectoproct.

**Figure 7.**
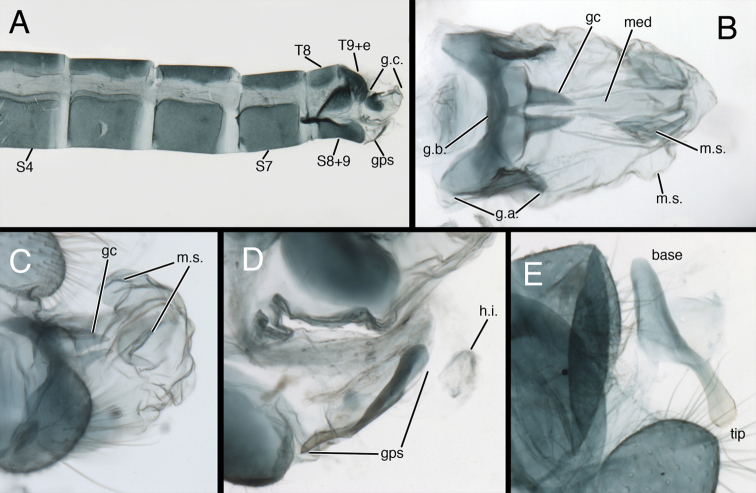
*Kymachrysa intacta* male abdomen. **A** Abdominal segments 4 to terminus (lateral), with genitalia everted **B** Gonarcal complex (dorsal) **C** Everted genitalia (dorsolateral), showing dorsal membranous sacs **D** Gonapsis (lateral), hypandrium internum (dorsal) **E** Gonapsis (ventral). **A, D** California, TRC **B, E** New York, TRC; **C** Colorado, CPG. *Abbreviations*: **gc** gonocornu **gps** gonapsis **g.a.** gonarcal apodeme **g.b.** gonarcal bridge **g.c.** gonarcal complex **h.i.** V-shaped hypandrium internum **med** mediuncus **m.s.** membranous sac **Sx** sternite, number **S8+9** fused eighth and ninth sternites **T8** eighth tergite **T9+e** fused ninth tergite and ectoproct.

**Figure 8.**
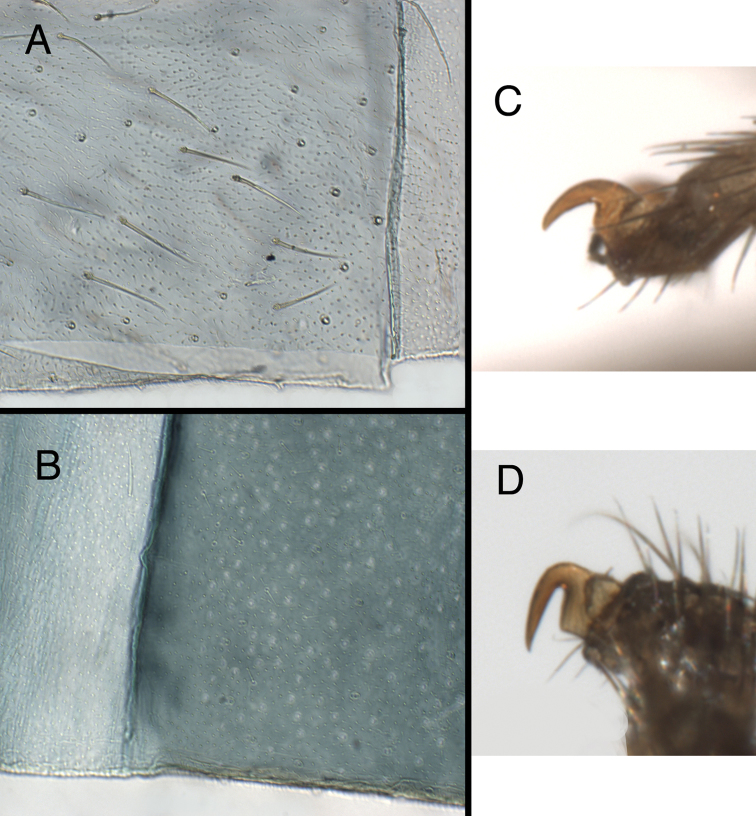
**A, B** Integument of fifth abdominal sternite. **A** *Kymachrysa placita*, without microtholi [typical of *Kymachrysa placita* and some *Kymachrysa intacta* populations (see text)] **B** *Kymachrysa intacta*, with microtholi (typical of some populations). **C, D** Prothoracic tarsal claw. **C** *Kymachrysa placita* **D** *Kymachrysa intacta*. **A** Type, Colorado, MCZ; **B** California, TRC; **C** Wyoming, SDNHM; **D** Minnesota, CMNH.

**Abdomen** (Figs 1C–F, 2A, B, 3A, B, 6A, 7A, 8A, B) with spiracles simple; callus cerci round, located dorsally on T9+ect, with trichobothria stemming from closely spaced sockets; ninth tergite (T9) and ectoproct fused, forming T9+ect; T9+ect completely divided dorsally.

**Male abdomen** (Figs 2, 6–8) slender, with sternites tall (~0.7× length), with or without microtholi; spiracles simple; terminal segments (A8–A9) compact; ectoproct with heavy apodeme along entire dorsal margin, terminus reaching proximal edge of eighth sternite (S8); eighth and ninth sternites fused; S8+9 shallow, with dorsal surface undulating, anterior section of S8+9 with heavy ventral apodeme extending proximally well beyond margin of segment, into S7; ventral surface indented mesally (hour-glass shaped). Gonapsis, hypandrium internum attached to membrane at tip of S9; gonapsis elongate, with basal margin rounded, toothed, distal margin expanded, curved; gonocristae absent. Gonarcus arcuate, with stout bridge, rounded, expanded lateral apodemes; gonocornua triangular, articulated on laterodistal margin of gonarcal bridge; base of gonocornua clear, tip dark, heavy, tapering to angulate terminus; entoprocessus absent. Mediuncus weak, comprising very lightly sclerotized, curved, mesal band distal to gonocornua, with trough-shaped dorsal surface, small, rounded, membranous terminus. Gonosaccus large, expanded dorsally as pair of large, eversible, distal sacs, expanded ventrally with a second pair of large, eversible sacs; gonosetae absent.

**Female abdomen** (Fig. 3) robust, not slender; terminalia compact. Praegenitale present, extending as truncated lobe from distal margin of S7, with distal, asymmetrical lobe, comb-shaped, with single long seta; lobe extending distally beyond S7 or curving internally. Colleterial gland, reservoir membranous, very delicate, extending proximally well into A7; transverse sclerite relatively large, broad, comb-shaped, with elongate teeth. Spermathecal complex simple; spermatheca small, pillbox shaped with small to moderate, U-shaped invagination, sail-like velum, opening to bursa copulatrix via elongate slit and small, membranous bursal duct; spermathecal duct elongate (>3× width of spermatheca), curvy, covered with fine hairs throughout (mature specimens), most dense, long distally, becoming short, stubby basally; immature specimens with basal ~10–20% of spermathecal duct smooth. Bursa copulatrix small, delicate, membranous, sac-like; bursal glands either absent or very small. Subgenitale large, robust, triangular in ventral view, with pair of distal lobes, extending outward or concealed beneath ectoprocts.

#### Etymology.

The prefix “*Kyma*-” comes from the Greek word *kýma* (*κύμα*), meaning wave, and refers to the wavy, or sinuate, longitudinal veins between the gradate veins of the forewings that distinguish the two species currently assigned to the genus. The suffix follows the traditional series of chrysopid names ending in “-*chrysa*” – Greek, feminine, “χρυσα” meaning golden.

#### Geographic distribution.

The genus, which currently includes only two species, appears to be restricted to North America (Canada, United States and Mexico, as far south as Mexico City) (Adams 1982, Penny et al. 1997, Tauber 2003, Valencia Luna et al. 2006, Garland and Kevan 2007, Freitas et al. 2009, Tauber and Flint 2010, all as *Ceraeochrysa* or *Chrysopodes*).

#### Characteristics of *Kymachrysa* larvae.

The larvae of only one of the *Kymachrysa* species (*Kymachrysa intacta*) were described (Tauber et al. 1998, as *Ceraeochrysa placita*). Later they were shown to share a large number of distinctive characteristics with the larvae of several species of *Chrysopodes*, and as a result the species was transferred to *Chrysopodes* (Tauber 2003, as *Chrysopodes placita*).

Recently, larvae from additional species of *Chrysopodes* were described in sufficient detail for more robust comparisons than were possible earlier. Now, detailed larval descriptions are available for six of the 47 currently recognized species of *Chrysopodes* [Tauber 2003: *Chrysopodes (Neosuarius) collaris* (Schneider); Silva et al. 2013: *Chrysopodes (Chrysopodes) divisus* (Walker), *Chrysopodes (Chrysopodes) fumosus* Tauber & Albuquerque, *Chrysopodes (Chrysopodes) geayi* (Navás), *Chrysopodes (Chrysopodes) linaefrons* Adams & Penny; *Chrysopodes (Chrysopodes) spinellus* Adams & Penny]. Comparisons with these species confirm that *Kymachrysa intacta* larvae differ only slightly from those of *Chrysopodes*. And, given the large percentage of species in both *Kymachrysa* and *Chrysopodes* with undescribed larvae, it is not clear at this time, which, if any, of these differences will be informative at the generic level.

**Figure 9.**
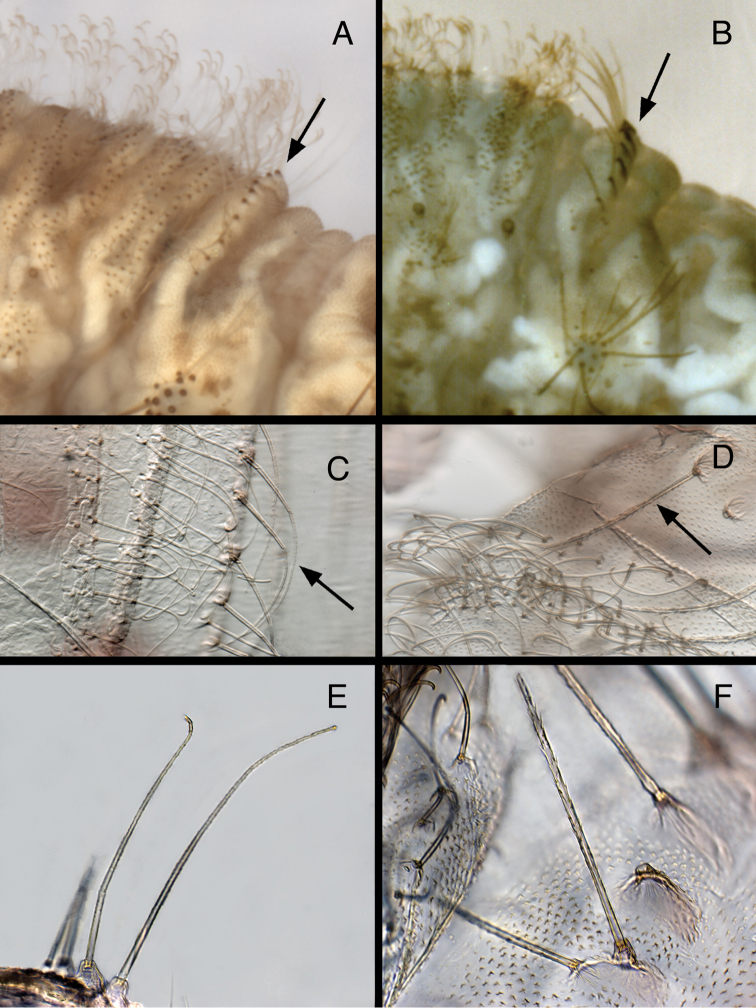
Larval characteristics that distinguish *Kymachrysa* from *Chrysopodes*. **A, B** Raised metathoracic posterior fold [Arrows indicate dark setal bases with distinctive anterior markings] **C–F** Setae on raised metathoracic fold [Note slender, curved structure, granular surface (*Kymachrysa*) vs robust, erect structure, thorny surface (*Chrysopodes*)] **A, C, E** *Kymachrysa intacta* (Californa, TRC) **B, D, F** *Chrysopodes geayi* (Rio de Janeiro, TRC).

Nevertheless, the following features appear most noteworthy:

(a) *Setae*: Previously, certain setae on the *Kymachrysa intacta* Semaphorant B were reported to be “serrated” or “thorny”, similar to those on *Chrysopodes*; these setae include the LS of the thorax and A4-A8, the large LDS on A6 and A7, and some dorsal thoracic setae (“serrated”: Tauber et al. 2000, as *Ceraeochrysa placita*; “thorny”: Tauber 2003, as *Chrysopodes placita*). Subsequent comparison of these setae with those of the six described *Chrysopodes* species (under higher magnification) indicates that the setal surface of *Kymachrysa intacta* (Semaphorant B) falls outside the range of variation exhibited by *Chrysopodes (Chrysopodes) * and *Chrysopodes (Neosuarius) *. Rather than serrated or thorny, the *Kymachrysa intacta* setae have a more sabulose (sandy) or granular surface (Fig. 9C–F). [Note: the same setae on Semaphorant A are thorny on both *Chrysopodes* and *Kymachrysa intacta*.]

(b) *Metathoracic fold*: One of the primary reasons for transferring *Kymachrysa intacta* to *Chrysopodes* (Tauber 2003, as *Chrysopodes placita*) was the shared characteristic of an unusual posterior fold on the larval metathorax. In both taxa, the fold rises well above the anterior part of the metathorax, and it bears a transverse row (R1) of robust setae that stem from enlarged chalazae and that are usually slightly longer than the submedian setae (SMS) on abdominal segments A1 through A6. However, subsequent comparisons indicated that aspects of this feature present some small, but significant differences between *Kymachrysa intacta* and *Chrysopodes*.

First, as discussed above, the surface of the R1 setae on *Kymachrysa intacta* Semaphorant B is distinctive. It is sabulose (sandy), not thorny as in *Chrysopodes*.

Second, although the body dimensions of the L3 larvae of *Chrysopodes (Chrysopodes) * and *Kymachrysa intacta* that we studied are similar, the length, robustness, and stiffness of the setae differ between the two taxa (Table 1, Fig. 9). For example, in *Chrysopodes (Chrysopodes) *, the R1 setae range in length between 0.28–0.42 mm, and they are thick and erect throughout their entire lengths. In comparison, the *Kymachrysa intacta* R1 setae range between 0.48–0.62 mm; they are slender throughout, and only the basal section stands erect – the distal section tends to curve. [Note: In the large bodied *Chrysopodes (Neosuarius) collaris* (Schneider), both the R1 setae and SMS are slightly longer and more slender and flexible than those of *Chrysopodes (Chrysopodes) * species (Table 1).]

**Table 1.** Larval setal lengths (range): comparison between *Kymachrysa intacta* and *Chrysopodes* species.

Species	T3-R1	SMS
*Kymachrysa intacta*	0.48–0.62 mm	0.28–0.43 mm
*Chrysopodes (Chrysopodes) divisus*	0.28–0.36 mm	0.24–0.36 mm
*Chrysopodes (Chrysopodes) geayi*	0.39–0.42 mm	0.21–0.36 mm
*Chrysopodes (Neosuarius) collaris*	0.43–0.51 mm	0.31–0.47 mm

Third, the larvae of *Chrysopodes* spp. (all instars, including the first) have dark brown markings on the frontal surface of the chalazae in metathoracic R1; these markings are elliptical to ovate and at least as broad as the setal base. In *Kymachrysa intacta* (second and third instars) they are light brown in color, elongate, and narrower than the setal base (Fig. 9A, B); they are either absent or very light in first instars.

#### Biological features of *Kymachrysa*.

Adult specimens of *Kymachrysa placita* are not common; those that we have seen were collected during July and August. No larval specimens are reported. *Kymachrysa intacta* appears to be more abundant; we have seen adult specimens collected from June through mid-October (mostly August), and we have collected larvae during March and April (overwintering second instars) and in September and October (prehibernal first instars).

**Figure 10.**
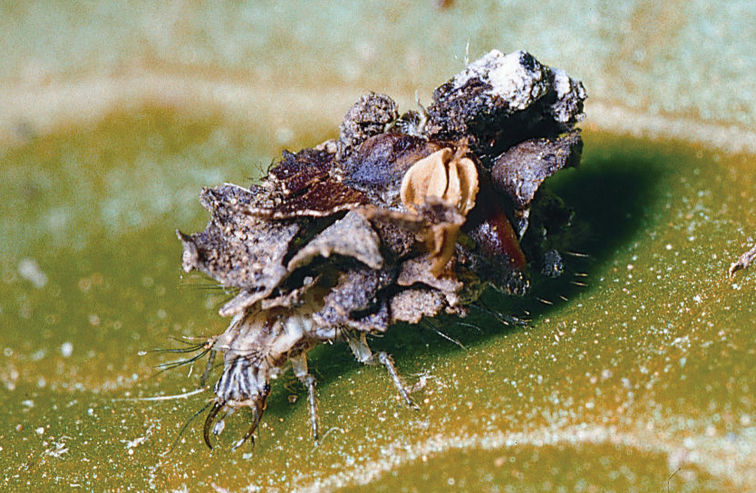
*Kymachrysa intacta* third instar (darkly marked). Note head markings, debris packet containing woody and dried plant material. (Photo: Stephen A. Marshall).

Biological features have been investigated only for *Kymachrysa intacta* (Tauber et al. 1998). In populations from both eastern and western USA (NY and CA), eggs are laid and larvae occur on the trunks of medium-sized to large-sized deciduous and evergreen trees. The larvae are debris-carriers; typically they carry pieces of bark or other woody or plant material that blends with their typical substrate (Fig. 10).

Developmental stages are relatively prolonged, and they are strongly influenced by photoperiod (Tauber et al. 1998). The life cycle appears to be univoltine. Larvae overwinter as diapausing second instars. Short daylengths decelerate development during the first instar, and they induce and maintain hibernal diapause in second instars. Daylength also may be important during the postdiapause developmental period. Under field conditions (Tompkins and Schyler Counties, NY), adults emerge in June, and eggs occur from July through late September or early October. Such a life cycle is unusual, but not unknown, for other chrysopids. For example, free-living second and third instars of *Pseudomallada* species also overwinter in a photoperiodically induced diapause (Principi and Sgobba 1987, 1993, Canard at al. 1990).

#### *Kymachrysa*’s generic relationships.

With the addition of *Kymachrysa*, a total of 17 genera of Chrysopini are now known from the New World. Table 2 lists the eleven that are reported from North America, including Mexico, and it provides references to their distributions.

**Table 2.** Genera in the tribe Chrysopini reported from North America, including the new genus, *Kymachrysa*.

*Ceraeochrysa* Adams, 1982 – New World, largely Neotropical; 15 species in North America (Valencia Luna et al. 2006, Garland and Kevan 2007, Freitas et al. 2009, Tauber and Flint 2010)
*Chrysopa* Leach in Brewster, 1815 – Holartic; eleven confirmed species in North America (Penny et al. 1997, Garland and Kevan 2007, Valencia Luna et al. 2006)
*Chrysoperla* Steinmann, 1964 – Worldwide; ten species from North America (Brooks 1994, Valencia Luna et al. 2006, Henry et al. 2012)
*Chrysopodes (Neosuarius) * Adams & Penny, 1985 – Neotropical; largely Central and South America; one species from North America (Adams and Penny 1985, Tauber 2003, Valencia Luna et al. 2006, Garland and Kevan 2007)
*Eremochrysa* Banks, 1903 – Nearctic, West Indies; largely southwestern USA & northern Mexico: *Eremochrysa (Chrysopiella) * Banks, 1911, four species; *Eremochrysa (Eremochrysa) * Banks 1903, thirteen species (Penny et al. 1997, Valencia Luna et al. 2006, Garland and Kevan 2007, Oswald 2013)
*Kymachrysa* gen. n. – Nearctic (Canada, USA, and montane Mexico), two species
*Meleoma* Fitch, 1855 – Nearctic, Neotropical, largely USA and Mexico; 28 species (Tauber 1969, Brooks and Barnard 1990, Penny 2006, Garland and Kevan 2007)
*Nineta* Navás, 1912 – Holarctic; two species from North America (USA, Canada) (Penny et al. 1997, Garland and Kevan 2007)
*Plesiochrysa* Adams, 1982 – Neotropical, Oriental, Australasia; ~25 species with two from North America (southern USA & Mexico) (Penny et al. 1997, Valencia Luna et al. 2006)
*Pseudomallada* Tsukaguchi, 1995 – Holarctic; large genus (~165 species) with five species from North America (Adams and Garland 1982)
*Yumachrysa* Banks, 1950 – Western USA, Mexico; four species (Penny et al. 1997, Oswald et al. 2002)

Above, we showed that *Kymachrysa* adults (males and females) express a number of characteristics that provide strong morphological support for a distinct genus. However, its relationship with other chrysopine genera remains perplexing. In general, the male genital structures resemble those of *Ceraeochrysa*, whereas the female genitalia (apart from the presence of a praegenitale in *Kymachrysa*) appear similar to those of several other genera (e.g., *Ungla*, *Pseudomallada*). Finally, its larval morphology is very close to that of *Chrysopodes*, and its biological traits (larval habitat, overwintering stage, photoperiodically controlled diapause) resemble those of *Pseudomallada*. Resolution of the dilemma posed by the above mixture of similarities awaits a broadly based phylogenetic analysis of chysopid genera.

### Modifications for Brooks and Barnard’s (1990) key to adults of chrysopid genera

In the most recent taxonomic key for chrysopid genera (Brooks and Barnard 1990), both *Kymachrysa* and *Ceraeochrysa* males are recovered at couplet 45. However, the two species can be differentiated with the following changes and additions to the couplet:

**Table:** 

45	Fore wing narrow (length: breadth > 2.8: 1); anal veins not crassate; radial crossveins (between R and Rs) usually straight; median fig [= gonarcal bridge] with dorsal horns [= gonocornua] (Brooks and Barnard 1990, fig. 268)	45A
–	Fore wing broad (length: breadth ≤ 2.8: 1); anal veins crassate; radial crossveins (between R and Rs) usually sinuate; median fig [= gonarcal bridge] without dorsal horns [= gonocornua] (Brooks and Barnard 1990, figs 335, 346)	*Chrysopodes* Navás
45A	Fore wing with veins between gradate veins straight; dorsum of T9+ect invaginated apically, but fused at least basally (male and female); male with ventral apodeme of S8+9 not elongated, extending anteriorly at most to margin of S8, not beyond; spermatheca cylindrical, with U-shaped or J-shaped bend opening to bursa copulatix	*Ceraeochrysa* Adams
–	Fore wing with veins between gradate veins sinuate (wavy); dorsum of T9+ect separate (unfused or divided) dorsally (male and female); male with ventral apodeme of S8+9 elongate, extending anteriorly beyond margin of S8; spermatheca pillbox-shaped, with velum opening to bursa copulatrix via small bursal duct	*Kymachrysa* gen. n.

### Catalog of *Kymachrysa* species

#### Kymachrysa placita

Taxon classificationAnimaliaNeuropteraChrysopidae

(Banks, 1908) comb. n.

Chrysopa placita Banks, 1908: 259 [MCZ, Lectotype, designated by Tauber and Flint 2010: 61].
Ceraeochrysa placita (Banks). First combination in *Ceraeochrysa* by Adams (1982: 73). Removed from *Ceraeochrysa* by Tauber (2003: 484). Combination reinstated by Freitas et al. (2009: 568), but subsequently considered uncertain by Tauber and Flint (2010: 64).
Chrysopodes (Neosuarius) placitus [= *placita*] (Banks). First combination in *Chrysopodes (Neosuarius) * by Tauber (2003: 484). Removed from *Chrysopodes* by Freitas et al. (2009: 568). Generic and subgeneric association with *Chrysopodes (Neosuarius) * considered uncertain by Tauber (2010: 12).
Chrysopa forreri Navás, 1913-14 [1914]: 97 [Syntype, The Natural History Museum, London (BMNH)]. Junior subjective synonym of *Ceraeochrysa placita* by Adams (1982: 73). Recognized as a junior subjective synonym of *Ceraeochrysa intacta* (Navás) by Tauber and Flint (2010: 62).
Chrysopa intacta Navás, 1912: 199 [Neotype, Canadian National Collection, Ottawa, (CNC), designated by Garland 1985: 138]. Junior subjective synonym of *Ceraeochrysa placita* by Garland (1985: 137). Recognized as a valid species, with uncertain generic assignment by Tauber and Flint (2010: 62).

##### Species specific characters.

The species was most recently re-described by Freitas et al. (2009: 568). Externally, it is recognized by its characteristic broad, dark brown facial markings (Fig. 4) and broadly cleft tarsal claws (Fig. 8C). The distinctive male features include: microtholi absent (Fig. 8A); ventral apodeme of S8+9 convex, with smooth curve; gonocornua broad, touching each other mesally (Fig. 6). Female character states include: spermathecal invagination small (< 1/4^th^ the width of the spermatheca), shallow (< 1/2 the depth of the spermatheca); basal section of spermathecal duct smooth, without hairiness (Fig. 3). The larvae and biology are unknown.

##### Geographic distribution.

We have seen specimens or reliable reports only from the USA (AZ: Chafee, Cochise Co.; CO: Clear Creek, Larimer, Jefferson Co.; NM: Cibola Co.; UT: Uintah Co.; WY: Albany Co.).

#### Kymachrysa intacta

Taxon classificationAnimaliaNeuropteraChrysopidae

(Navás, 1912) comb. n.

Chrysopa intacta Navás, 1912: 199 [Original syntype reported to have been retained in Navás collection, probably destroyed; Neotype, CNC; designated by Garland (1985: 137)]. Junior subjective synonym of *Ceraeochrysa placita* by Garland (1985: 137).
Ceraeochrysa intacta (Navás), genus *incertae sedis*. Recognized as a valid species, with uncertain generic assignment by Tauber and Flint (2010: 62).
Chrysopa forreri Navás, 1913-14 [1914]: 97 [Syntype, BMNH]. Junior subjective synonym of *Ceraeochrysa intacta* by Tauber and Flint (2010: 61).
Ceraeochrysa chiricahuae Freitas and Penny, in Freitas et al. 2009: 594 [Holotype, CAS]. Junior subjective synonym of *Ceraeochrysa intacta* by Tauber and Flint (2010: 61).

##### Remarks.

In addition to the citations listed above that refer to synonymies and nomenclatural changes, Garland and Kevan (2007: 59) provide a list of references containing information on *Kymachrysa intacta* under the name *placita*/*placitus* [as *Chrysopa*, *Ceraeochrysa*, *Chrysopodes (Neosuarius) *, *Oviedus*]; other references include Tauber and de León (2001, as *Ceraeochrysa placita*); Tauber (2010: 12, as *Chrysopodes placitus*); Tauber et al. 2014: Suppl, Material, as *Ceraeochrysa placita*, *incertae sedis*).

##### Species specific characters.

This species was re-described by Tauber et al. (1998, 2000, as *Ceraeochrysa placita*), Tauber (2003, as *Chrysopodes placita*), and Freitas et al. (2009: 594, as *Ceraeochrysa chiricahuae*). Adults are recognized by their slender, red markings on the head and thorax (Fig. 5) and narrowly cleft tarsal claws (Fig. 8D; also see Garland 1982, fig. 74k), as well as their distinctive male and female genitalia. The male features include: microtholi sometimes present (see below); ventral apodeme of S8+9 irregular, with several small curves and a mesal bend; gonocornua narrow, separated from each other basally (Fig. 7). In the female, the spermathecal invagination is large (~1/3^rd^ the width of the spermatheca) and relatively deep (> ½ the depth of the spermatheca), and the basal section of the spermathecal duct is hairy (Fig. 3).

Specimens of *Kymachrysa intacta* from eastern and western North America appear very similar to each other, but we found some geographic variation of note. First, the male abdominal sternites are densely covered with microtholi on our specimens from California (Alameda, Kern, and Sierra Counties) (Fig. 8B). In contrast, microtholi are very sparse or absent on specimens from Utah (Wasatch County) and Colorado (Larimer County), and absent from specimens from New Hampshire (Belknap County) and New York (Tompkins and Schuyler Counties) (as in Fig. 8 for *Kymachrysa placita*; also see Garland 1982). Second, larvae (second and third instars) from the west were reported to have somewhat denser abdominal setation than those of the east (Tauber et al. 1998). And, finally, the length of the stalk that supports the egg is considerably longer in eastern than in western populations (eastern: 2.1–3.3 mm; western: 1.4–2.3 mm) (Tauber et al. 1998). Given the potential role of microtholi in courtship, we suspect that the above variation may indicate at least some reproductive isolation between eastern and western populations.

**Figure 11.**
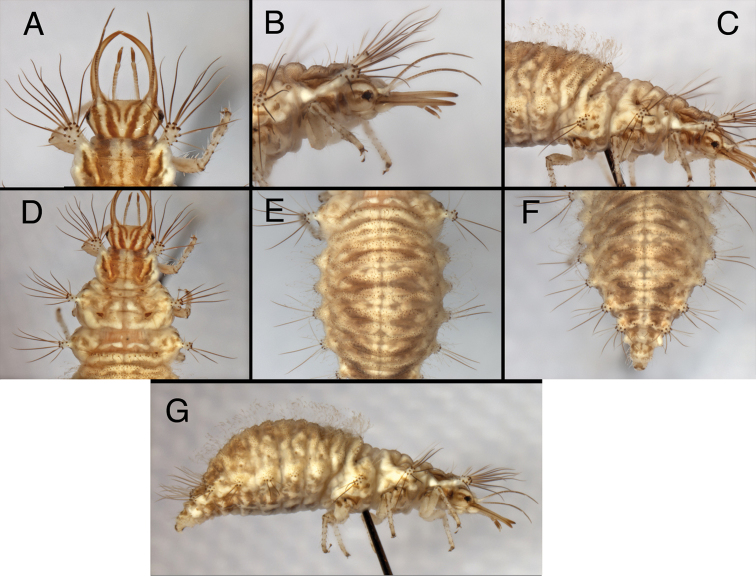
*Kymachrysa intacta* third instar. **A** Head (dorsal) **B** Head (lateral) **C** Thorax (lateral) **D** Thorax (dorsal) **E** Abdominal segments 1–4 (dorsal) **F** Abdominal segments 4–10 (dorsal) **G** Body (lateral). All New York, TRC.

The larvae of *Kymachrysa intacta* have been described (Tauber et al. 1998, 2000, Tauber 2003); for images, see Fig. 11 here. Below are some modifications and corrections to the earlier descriptions:

(a) Semaphorant A (L1): Thorax as figured by Tauber et al. (1998, Fig. 8), with following differences: Mesothorax with Sc1, Sc2 present anteriorly on folds as in *Chrysopodes* (see Tauber 2003, fig. 10); Sc3 present, posteromesal to lateral tubercles, with one medium-length and sometimes one small associated setae; S1 small or absent; S2 medium-length, mesal to Sc3, smooth to finely sabulose. Metathorax with Sc3 present, mesal to lateral tubercle, with one or two small associated setae (S1Sc3, S2Sc3); several very small setae anterior to Sc3, probably associated with a very pale Sc2.

(b) Semaphorant B (L3, L2): Prothorax with S1 and S1Sc1 smooth to very finely sabulose, not thorny. Mesothorax with Sc2 present mesal to lateral tubercles, with two small associated setae (S1Sc2, S2Sc2); S1 present, small; S2 small or absent; S3 of medium-length, slightly granular; S4 small, smooth; but S1Sc3 small, S2Sc3 slightly longer. Metathorax with posterior row of setae (R1) arising from large chalazae.

##### Geographic distribution.

This species occurs broadly throughout North America. We have seen specimens or reliable records from: Canada (ON, QC; see Garland 1984); United States (AZ, CA, CO, MN, MO, NC, NH, NM, NV, NY, OR, TN, TX, UT, WS, WV, WY; see Penny et al. 1997); and Mexico (Dgo., D.F., Mich.; see Valencia Luna et al. 2006).

*Kymachrysa placita* and *Kymachrysa intacta* were collected sympatrically in Rustler Park, Cochise Co., AZ, 6-Aug-1991, by R. & J. Robertson (CAS).

## Supplementary Material

XML Treatment for Kymachrysa

XML Treatment for Kymachrysa placita

XML Treatment for Kymachrysa intacta
